# Molecular and Pathologic Characterization of YAP1-Expressing Small Cell Lung Cancer Cell Lines Leads to Reclassification as SMARCA4-Deficient Malignancies

**DOI:** 10.1158/1078-0432.CCR-23-2360

**Published:** 2023-12-07

**Authors:** Jin Ng, Ling Cai, Luc Girard, Owen W.J. Prall, Neeha Rajan, Christine Khoo, Ahida Batrouney, David J. Byrne, Danielle K. Boyd, Ariena J. Kersbergen, Michael Christie, John D. Minna, Marian L. Burr, Kate D. Sutherland

**Affiliations:** 1 ACRF Cancer Biology and Stem Cells Division, The Walter and Eliza Hall Institute of Medical Research, Parkville, Victoria, Australia.; 2 Department of Medical Biology, The University of Melbourne, Parkville, Victoria, Australia.; 3 Quantitative Biomedical Research Center, Peter O'Donnell Jr. School of Public Health, UT Southwestern Medical Center, Dallas, Texas.; 4 Children's Research Institute, UT Southwestern Medical Center, Dallas, Texas.; 5 Simmons Comprehensive Cancer Center, UT Southwestern Medical Center, Dallas, Texas.; 6 Hamon Center for Therapeutic Oncology Research, UT Southwestern Medical Center, Dallas, Texas.; 7 Department of Anatomical Pathology, Peter MacCallum Cancer Centre, Melbourne, Victoria, Australia.; 8 Department of Anatomical Pathology, The Royal Melbourne Hospital, Parkville, Victoria, Australia.; 9 Personalised Oncology Division, The Walter and Eliza Hall Institute of Medical Research, Parkville, Victoria, Australia.; 10 Department of Internal Medicine, UT Southwestern Medical Center, Dallas, Texas.; 11 Department of Pharmacology, UT Southwestern Medical Center, Dallas, Texas.; 12 Division of Genome Science and Cancer, The John Curtin School of Medical Research, The Australian National University, Canberra, Australian Capital Territory, Australia.; 13 Department of Anatomical Pathology, ACT Pathology, Canberra Health Services, Canberra, Australian Capital Territory, Australia.; 14 Sir Peter MacCallum Department of Oncology, University of Melbourne, Victoria, Australia.

## Abstract

**Purpose:**

The classification of small cell lung cancer (SCLC) into distinct molecular subtypes defined by ASCL1, NEUROD1, POU2F3, or YAP1 (SCLC-A, -N, -P, or -Y) expression, paves the way for a personalized treatment approach. However, the existence of a distinct YAP1-expressing SCLC subtype remains controversial.

**Experimental Design:**

To better understand YAP1-expressing SCLC, the mutational landscape of human SCLC cell lines was interrogated to identify pathogenic alterations unique to SCLC-Y. Xenograft tumors, generated from cell lines representing the four SCLC molecular subtypes, were evaluated by a panel of pathologists who routinely diagnose thoracic malignancies. Diagnoses were complemented by transcriptomic analysis of primary tumors and human cell line datasets. Protein expression profiles were validated in patient tumor tissue.

**Results:**

Unexpectedly, pathogenic mutations in *SMARCA4* were identified in six of eight SCLC-Y cell lines and correlated with reduced SMARCA4 mRNA and protein expression. Pathologist evaluations revealed that SMARCA4-deficient SCLC-Y tumors exhibited features consistent with thoracic SMARCA4-deficient undifferentiated tumors (SMARCA4-UT). Similarly, the transcriptional profile *SMARCA4*-mutant SCLC-Y lines more closely resembled primary SMARCA4-UT, or SMARCA4-deficient non–small cell carcinoma, than SCLC. Furthermore, SMARCA4-UT patient samples were associated with a YAP1 transcriptional signature and exhibited strong YAP1 protein expression. Together, we found little evidence to support a diagnosis of SCLC for any of the YAP1-expressing cell lines originally used to define the SCLC-Y subtype.

**Conclusions:**

SMARCA4-mutant SCLC-Y cell lines exhibit characteristics consistent with SMARCA4-deficient malignancies rather than SCLC. Our findings suggest that, unlike ASCL1, NEUROD1, and POU2F3, YAP1 is not a subtype defining transcription factor in SCLC.

*See related commentary by Rekhtman, p. 1708*

**Translational Relevance**

In this study, we perform an in-depth characterization of the YAP1-expressing human SCLC cell lines, utilized widely for studying subtype-specific vulnerabilities in SCLC. Genomic analyses identified an association between SCLC-Y cell lines and pathogenic alterations in *SMARCA4*. Morphologic and molecular evaluations performed with *SMARCA4*-mutant SCLC-Y lines revealed inconsistencies with an SCLC diagnosis. Rather, *SMARCA4*-mutant SCLC-Y cells represent SMARCA4-deficient undifferentiated tumors (SMARCA4-UT), a recently described lung malignancy that can mimic SCLC. Importantly, we show that primary human SMARCA4-UT also express high levels of YAP1. Our results suggest that YAP1 is not a reliable subtype defining transcription factor for SCLC. Importantly, cell lines commonly used as preclinical models of YAP1-expressing SCLC are in fact SMARCA4-UT, for which to date there has been a lack of representative human disease models. Thus, these preclinical models previously thought to be SCLC now represent a new preclinical model resource for study of SMARCA4-UT.

## Introduction

Small cell lung cancer (SCLC) is the deadliest form of lung cancer with limited durable responses to platinum-based chemotherapy and immune checkpoint blockade-based immunotherapy (1). Unlike lung adenocarcinoma, where the development of therapies targeting specific driver oncogenes has enabled genetic alteration-based stratification, recurrent targetable mutations have not been identified in SCLC. The recent classification of SCLC into four molecular subtypes based on transcription factor expression therefore represents a major advance, providing a new paradigm to facilitate better understanding of SCLC biology and a path towards the development of molecularly targeted treatments (2, 3). These four molecular subtypes are defined by the dominant expression of *ASCL1* (SCLC-A), *NEUROD1* (SCLC-N), *POU2F3* (SCLC-P), or *YAP1* (SCLC-Y). Indeed, recent studies characterizing human SCLC cell lines have identified several subtype-specific therapeutic vulnerabilities (4). For example, Delta-like ligand 3 (DLL3), an inhibitory Notch ligand, is the target of a plethora of clinical therapeutics in SCLC and is reported to exhibit high expression in SCLC-A tumors (5).

The seminal initial classification of SCLC subtypes was based on RNA-sequencing data from SCLC cell line data in the Cancer Cell Line Encyclopedia (CCLE; ref. 2) and deposited data from two well-characterized SCLC patient cohorts [Rudin and colleagues (6) and George and colleagues (7)]. Although the SCLC-A, SCLC-N, and SCLC-P groups within this classification are comprised of both primary tumors and cell lines, notably the SCLC-Y group consists mostly of cell lines (6). Furthermore, although a follow-up study performing immunohistochemistry (IHC) in a well-characterized patient cohort validated the presence of NEUROD1, ASCL1, and POU2F3 expressing primary SCLC tumors, a distinct YAP1 expressing SCLC subtype was not identified (8). Nevertheless, YAP1 expressing SCLC cells have been detected in patient-derived xenografts, genetically engineered mouse (GEM) models, and some patient cohorts (9–11). In most cases, YAP1 expression in these settings was focal or heterogenous, although rare YAP1 dominant primary SCLC tumors have been reported (12). Together, these observations have fuelled the controversy around the existence of SCLC-Y as a *bona fide* SCLC subtype (8, 11, 13).

YAP1 expressing SCLC cells are characterized by low expression of classical neuroendocrine markers synaptophysin, chromogranin A, NCAM1 (CD56), and INSM1. Although substantial expression of classic neuroendocrine markers helps validate a SCLC diagnosis, 5% to 10% of SCLC primary tumors may show low or absent neuroendocrine marker expression (14). Thus, a histologic diagnosis of SCLC can be made in the absence of positive neuroendocrine marker IHC if the tumor morphology is characteristic (15). Nevertheless, neuroendocrine marker low/negative SCLC presents a clinical diagnostic challenge as there are several other malignancies that can pathologically mimic SCLC, such as basaloid squamous cell carcinoma and poorly differentiated adenocarcinoma, which must be excluded (14). Importantly, many of the tumors that can mimic SCLC express YAP1, which is an additional complicating factor to the use of YAP1 as a defining factor for SCLC. Because there are standard treatment regimens applied when a patient receives a clinical/pathologic diagnosis of SCLC, it is of substantial importance to be sure of this diagnosis when lung tumors posing diagnostic problems such as a YAP1 expressing, neuroendocrine marker low expressing tumor are encountered.

Although the role of YAP1 as a subtype defining transcription factor for SCLC remains controversial, the existence of a subset of SCLC tumors that lack expression of NEUROD1, ASCL1, or POU2F3 and characteristically exhibit low expression of neuroendocrine markers is well recognized and has been reproducibly identified in several SCLC patient cohorts (8, 12, 13). This subset of neuroendocrine low and A/N/P negative SCLC tumors has become particularly significant following the recognition that these tumors typically express higher levels of inflammation-associated genes and have increased T-cell infiltration compared with “classical” neuroendocrine high SCLC and thus are potentially the most likely to benefit from immune checkpoint blockade immune therapy (13, 16). In some studies these A/N/P negative tumors have been termed “triple negative,” while other studies have termed them neuroendocrine low SCLC with an “inflamed” gene signature “SCLC-I.” Importantly, retrospective stratification of patients with SCLC in the IMpower133 clinical trial demonstrates that patients with SCLC-I tumors show increased survival following chemoimmunotherapy with atezolizumab (anti-PD-L1) and etoposide, compared with SCLC-A, -N, or -P (13).

Given the apparent increased responsiveness of SCLC-I subtype tumors to immunotherapy, there is a critical need to better understand the origin and biology of these tumors. Notably, overlapping features of low neuroendocrine marker expression and an “inflamed” gene signature in cell lines defined as SCLC-Y has meant that these lines have frequently been used as models of non-neurodocrine, “inflamed” or “triple negative” SCLC (17). However, the relationship between SCLC-I and the initially defined SCLC-Y subtype, and whether these YAP1 positive SCLC-Y cell lines present a reliable model of “neurodocrine low” SCLC, has not been clearly defined. This is of particular significance given that YAP1 expression has been reported to be an unreliable marker of SCLC-I in clinical cohorts (13, 18). To better understand the biology and origins of SCLC-Y, we therefore performed a comprehensive molecular and histologic characterization of the tumor lines used to define this proposed subtype of SCLC.

## Materials and Methods

### Cell culture and transplantation studies

The following human SCLC cell lines were purchased from ATCC: NCI-H69 (RRID:CVCL_1579), NCI-H1092 (RRID:CVCL_1454), NCI-H2227 (RRID:CVCL_1542), NCI-H2171 (RRID:CVCL_1536), NCI-H1694 (RRID:CVCL_1489), NCI-H211 (RRID:CVCL_1529), NCI-H1048 (RRID:CVCL_1453), NCI-H1341 (RRID:CVCL_1463), SW1271 (RRID:CVCL_1716), NCI-H841 (RRID:CVCL_1595), DMS114 (RRID:CVCL_1174), NCI-H196 (RRID:CVCL_1509), NCI-H661 (RRID:CVCL_1577), and NCI-H1581 (RRID:CVCL_1479). Human SCLC cell line SBC5 (RRID:CVCL_1679) was obtained directly from Cell Bank Australia. Human SCLC cell lines NCI-H82 (RRID:CVCL_1591), NCI-H446 (RRID:CVCL_1562), and NCI-H146 (RRID:CVCL_1473) were obtained from Dr. Marian L. Burr (The John Curtin School of Medical Research) and NCI-H526 (RRID:CVCL_1569) was obtained from Dr. David Huang (Walter and Eliza Hall Institute). All cell lines were cultured according to the manufacturer's recommendations with routine PCR-based mycoplasma testing every 3 months. All cell lines were authenticated by short tandem repeat (STR) profiling from the cell banks they were originally purchased from and by the Australian Genome Research Facility (Melbourne). The cell lines in this study were cultured for fewer than 6 months after receipt. Tumor xenografts were generated by subcutaneous injection of 1 × 10^6^ cells in 50% growth factor reduced Matrigel (BD Biosciences) into the flanks of CBA.Nude mice and harvested for paraffin embedding once tumors reached a volume of 200 to 1,000 mm^3^. All animal experiments were conducted according to the regulatory standards approved by the Walter and Eliza Hall Institute Animal Ethics Committee.

### Histology and IHC

Subcutaneous tumors were harvested and fixed in 10% (v/v) neutral-buffered formalin at room temperature or 4% (w/v) paraformaldehyde at 4°C for at least 24 hours. Fixed samples were dehydrated, cleared, and embedded in paraffin. Sections 3 μm thick were stained with haematoxylin and eosin (H&E) and sections 4-μm thick were immunostained. The following antibodies for IHC were purchased from Abcam: anti-SMARCA4 (#ab110641:RRID:AB_10861578), anti-synaptophysin (#ab32127:RRID:AB_2286949), anti-Claudin 4 (#ab53156:RRID:AB_869176), and anti-wide-spectrum cytokeratin (#ab9377:RRID:AB_307222). The following antibodies were purchased from Cell Signaling Technology: anti-RB1 (#9309:RRID:AB_823629), anti-YAP1 (#14074: RRID:AB_2650491), and anti-SMARCA2 (#11966:RRID:AB_2797783). Anti-CD56 was purchased from Millipore Sigma (#AB5032:RRID:AB_2291692), anti-INSM1 was purchased from Santa Cruz Biotechnology (#sc271408:RRID:AB_10607955), anti-TTF1 was purchased from Leica Biosystems (#NCL-TTF-1:RRID:AB_442138), anti-p40 was purchased from Biocare Medical (#ACI3066 A, C: RRID:AB_2858274), and Roche (#790–4950: RRID:AB_2935820). A complete list of antibody dilutions, antigen retrieval conditions, and detection solutions are detailed in Supplementary Table S1.

Primary antibodies (SMARCA4, INSM1, TTF1, p40, and RB1) were stained using the Roche Ventana BenchMark ULTRA automated staining system. While staining with primary antibodies (CD56, synaptophysin, wide-spectrum cytokeratin, YAP1, SMARCA2, and Claudin 4) was performed on either the Dako Omnis or Leica Bond automated staining system, using standard protocols. Stained xenografts were evaluated by M.L.B. for either nuclear, membranous, or cytoplasmic staining and *H* scores were calculated as described previously (Supplementary Table S1; ref. 8).

### Pathology assessment of cell line xenografts

Five anatomical pathologists who routinely diagnose thoracic malignancies across different hospitals within Australia were recruited. Prior to examination of the cell line xenografts, pathologists were provided with the same clinical history for all slides: 60-year-old male with a lung mass and mediastinal lymphadenopathy.

Pathologists were first asked to assess the H&E xenografts, in a blinded fashion, and provide a provisional diagnosis, as a “yes” or “no,” of whether the H&E appearances of each xenograft were consistent with a pathologic diagnosis of SCLC. Following this provisional diagnosis, the pathologists were provided with images of IHC staining and a table (Supplementary Table S2) summarizing the immunophenotype of each xenograft together with a link to the *Thoracic SMARCA4-deficient undifferentiated tumor* chapter in the 5th Edition of WHO Classification of Tumors (15). Pathologic assessment of each xenograft was performed independently with each pathologist blinded to the provisional and re-evaluated diagnosis from the other pathologists.

### Ethics and patient samples

All procedures performed in this study involving patient material were in accordance with the ethical standards of the institutional research committees and with the Declaration of Helsinki. This study was approved by the Walter and Eliza Hall Institute Human Research Ethics Committee (#22/7), Australian Capital Territory (ACT) Health Human Research Ethics Committee (2022.LRE.00216, 2022/ETH02563) and Peter MacCallum Cancer Centre Human Research Ethics Committee (HREC no.: 03/90). Tumor samples from patients diagnosed with SMARCA4-UT were identified through review of pathology records at Peter MacCallum Cancer Centre and Canberra Hospital. The clinical profiles for all SMARCA4-UT patients such as sex and age at diagnosis are summarized in Supplementary Table S3. Clinical profiles for Case 1, Case 2, and Case 3 were not collected as the ethics under which these samples were collected did not permit for this. In addition, the results of the diagnostic immunohistochemical panel performed for these samples are summarized in Supplementary Table S3. H&E stained slides and IHC were reviewed by pathologists involved in this study to verify the original diagnosis of SMARCA4-UT and sections from stored FFPE samples were additionally stained for YAP1 (Supplementary Table S1). H&E scans of the patient biopsy blocks from which cell lines NCI-H661 and NCI-H1581 were derived, were acquired through UTSW.

### Multi-omics analysis of cell line and patient samples

SCLC class was determined by CCLE RNA-seq (19, 20) or the separate SCLC molecular dataset for RNA-seq and whole-exome sequencing performed by the Hamon Center (deposited at dbGaP Study Accession: phs001823.v1.p1; https://www.ncbi.nlm.nih.gov/projects/gap/cgi-bin/study.cgi?study_id=phs001823.v1.p1) and cited in Cai and colleagues (16) data for driver transcription factors; *ASCL1*, *NEUROD1*, *POU2F3*, and *YAP*. For each cell line, the transcription factor with the highest expression among the four is used to assign the transcription factor class. These annotations were concordant with that described by Rudin and colleagues (2) except for one cell line (H2227), which was annotated SCLC-A using the UTSW dataset (Supplementary Table S4). Mutations present in over 50% of cell lines within each molecular subtype were extracted via cBioportal.org (21, 22). Known pathogenic mutations were annotated using OncoKB (RRID:SCR_014782) and https://cancerhotspots.org available through cBioportal.org (RRID:SCR_014555).

We used the following datasets from UT Lung SPORE: microarray, RNA-seq gene expression data and mutation data (dbGAP Study Accession No.: phs001823.v1.p1). We used the following datasets from DepMap (19): RNA-seq(CCLE_depMap_19Q1_TPM.csv), microarray (CCLE_Expression_Entrez_2012–09–29.gct), proteomics (Table_S2_Protein_Quant_Normalized.xlsx; ref. 20), mutation (downloaded as CCLE_DepMap_18q3_maf_20180718.txt), and structural variation (CCLE_translocations_SvABA_20181221.xlsx) datasets. For each cell line, we computed the neuroendocrine (NE) score as described previously (23), but with the updated NE signature based on RNA-seq data (16). Le Loarer datasets (24) were downloaded from SRA under accession no. SRP052896 and processed using the RNA-seq pipeline (https://git.biohpc.swmed.edu/BICF/Astrocyte/rnaseq) from UTSW Bioinformatics Core Facility. The CCLE cell lines annotated as SCLC, NSCLC, or SCCOHT was merged with the Le Loarer RNA-seq data and were quantile normalized. We used a previously published YAP1 SCLC gene signature (10) for unsupervised hierarchal clustering of CCLE cell lines and Le Loarer patient samples.

To analyze mRNA expression of markers associated with SMARCA4 and a neuroendocrine phenotype, CCLE cell lines were grouped into “SMARCA4-UT cluster” (*n* = 7), “SMARCA4 mutant NSCLC” (*n* = 31), “SMARCA4 proficient NSCLC” (*n* = 88), and “SCLC-A, -N, -P” (*n* = 43). See Supplementary Table S5 for a full annotated list of cell lines. Gene expression (*Z*-score; log RNAseq RPKM) values were accessed through cBioportal.org (21, 22).

### Statistical analysis

All statistical analyses were conducted in Graph Pad Prism Version 9 (RRID:SCR_002798) or R studio Version 4.0.2 (RRID:SCR_001905). Data were assessed for normal distribution using a Shapiro–Wilk test. Data were considered normally distributed if the *P*-value was > 0.05. If the data were not normally distributed, a Kruskal–Wallis test with a Dunn's multiple comparison test (between group) was conducted for multiple groups, or a Mann–Whitney test comparing tanks between two groups. The level of significance was set to *P* < 0.05.

### Data availability

The DNA and mRNA expression sequencing data generated by UTSW are available in Supplementary Table S4. The NE score for SCLC cell lines is available in Supplementary Table S6. The data analysed in this study were obtained from Sequence Read Archive (SRA; RRID:SCR_004891) at SRP052896 (https://www.ncbi.nlm.nih.gov/sra?term=SRP052896); from database of Genotypes and Phenotypes (dbGaP) (RRID:SCR_002709) at phs001823.v1.p1 (https://www.ncbi.nlm.nih.gov/projects/gap/cgi-bin/study.cgi?study_id=phs001823.v1.p1); from Dependency Map (DepMap) (RRID:SCR_017655) at 19Q1 (https://depmap.org/portal/download/all/?releasename=DepMap+Public+19Q1), 18Q3 (https://depmap.org/portal/download/all/?releasename=DepMap+Public+18Q3), CCLE2019 (https://depmap.org/portal/download/all/?releasename=CCLE+2019); from the Broad Institute (RRID:SCR_013836) at CCLE Legacy Data (https://data.broadinstitute.org/ccle_legacy_data/mRNA_expression/); and from original articles Nusinow and colleagues (20) at Supplementary Table S2.

## Results

### Pathogenic *SMARCA4* mutations are enriched in SCLC-Y

**Figure 1.**
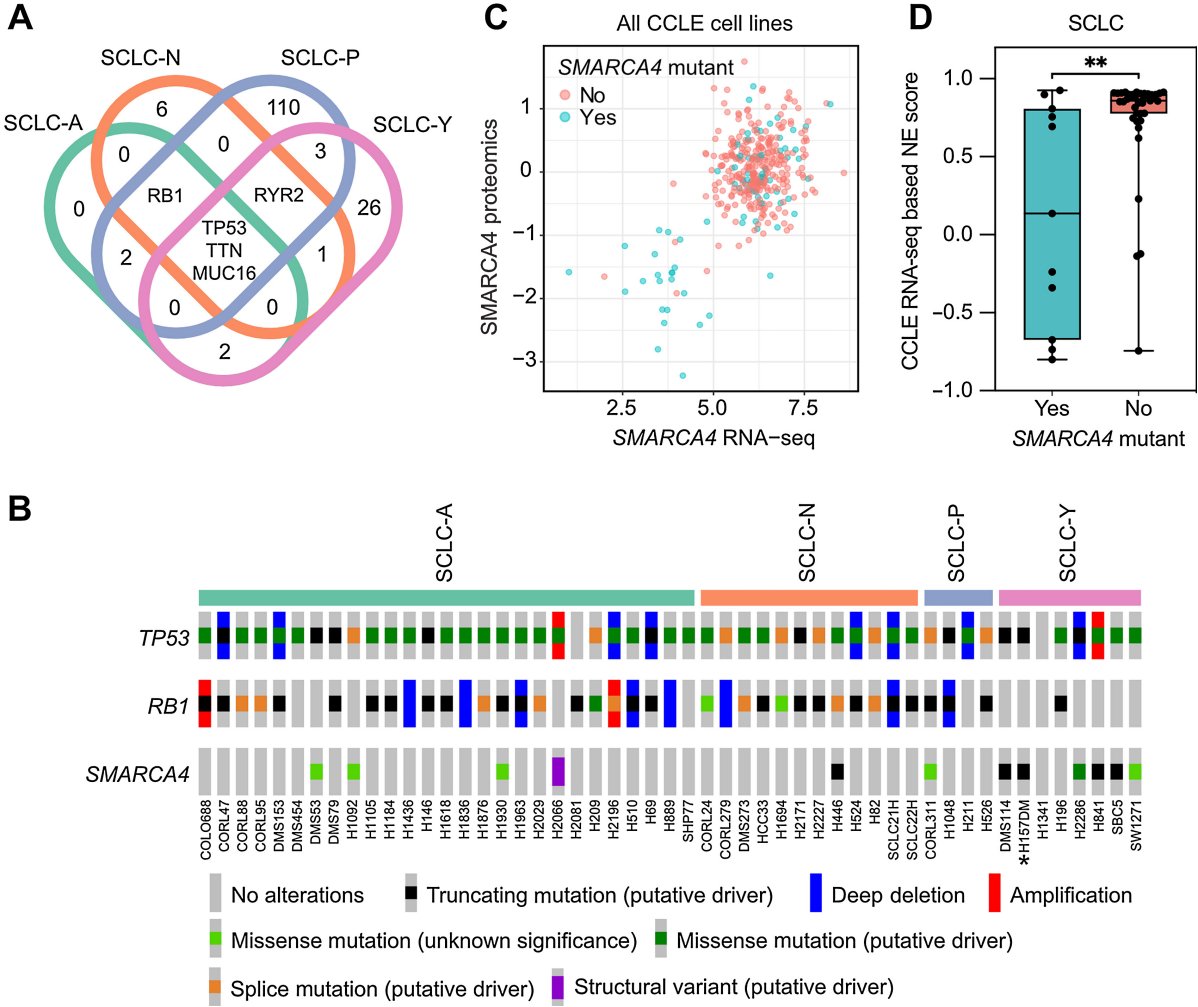
*SMARCA4* mutations are enriched within SCLC-Y cell lines. **A,** Venn diagram showing co-occurring and subtype-specific mutations associated with each SCLC molecular subtype. Genetic mutations that are present in at least 50% of samples within each SCLC subtype based on Rudin and colleagues (2) were identified through cBioportal. There are 26 mutations exclusive to SCLC-Y cell lines (Supplementary Table S7). **B,** Of all 26 mutations identified, *SMARCA4* mutations were present in six of eight SCLC-Y cell lines, together with *TP53* mutations on an *RB1* wild-type background. *, H157DM was previously annotated in CCLE as H1339. **C,** All cell lines in the CCLE (lung and nonlung cancer cell lines) that had complete proteomic and RNA-seq profiles were interrogated for the correlation between SMARCA4 protein and mRNA. There is a positive correlation (Pearson correlation = 0.53; *P* = 1.5e−27) between low *SMARCA4* mRNA and loss of SMARCA4 protein. **D,** SCLC cell lines with *SMARCA4* mutations have a significantly lower NE-score compared with wild-type (WT) *SMARCA4* SCLC cell lines (Mann–Whitney test; **, *P* = 0.0068). SCLC cell lines in the CCLE were binned into *SMARCA4* mutant and WT groups. NE scoring was performed using a previously published 50-gene transcriptomic signature (16).

SCLC almost universally harbors mutations of both *TP53* and *RB1*, unusually however, SCLC-Y cell lines frequently lack *RB1* mutations (25). We therefore interrogated the mutational landscape of 50 SCLC cell lines used to classify the SCLC-Y subtype through the Cancer Cell Line Encyclopaedia (CCLE) and Hamon Center genomic datasets (19) and identified 26 frequently mutated genes unique to SCLC-Y cell lines (Fig. 1A; Supplementary Table S7). This dataset included all eight YAP1 expressing SCLC lines previously included in the classification of the SCLC-Y lineage (2). Notably, mutations in *SMARCA4* (also known as *BRG1*), which encodes an ATPase subunit of SWI/SNF chromatin-remodeling complexes (26), were observed in six out of eight SCLC-Y lines and found mutually exclusive with *RB1* mutations (Fig. 1B; Supplementary Fig. S1A). All cancer cell lines harbouring frame shift or nonsense mutations in *SMARCA4* showed reduced SMARCA4 mRNA and protein abundance (Fig. 1C; Supplementary Fig. S1B), consistent with the *SMARCA4* “class 1 alterations” observed in non–small cell lung carcinoma (NSCLC; ref. 27). All six *SMARCA4*-mutant SCLC-Y lines also harboured *TP53* mutations, but other genomic alterations previously observed to co-occur with *SMARCA4*-mutant NSCLC, such as *STK11*, *KEAP1*, and *KRAS*, were not identified (Supplementary Table S8). Furthermore, in line with the specific enrichment of *SMARCA4* mutations in SCLC-Y tumors, *SMARCA4*-mutant SCLC cell lines exhibited significantly lower expression of a previously defined neuroendocrine (NE) transcriptomic signature (16, 23) compared with *SMARCA4* wildtype SCLC cell lines (Fig. 1D; Supplementary Table S6).

### Histopathologic classification of SMARCA4-deficient SCLC-Y

**Figure 2.**
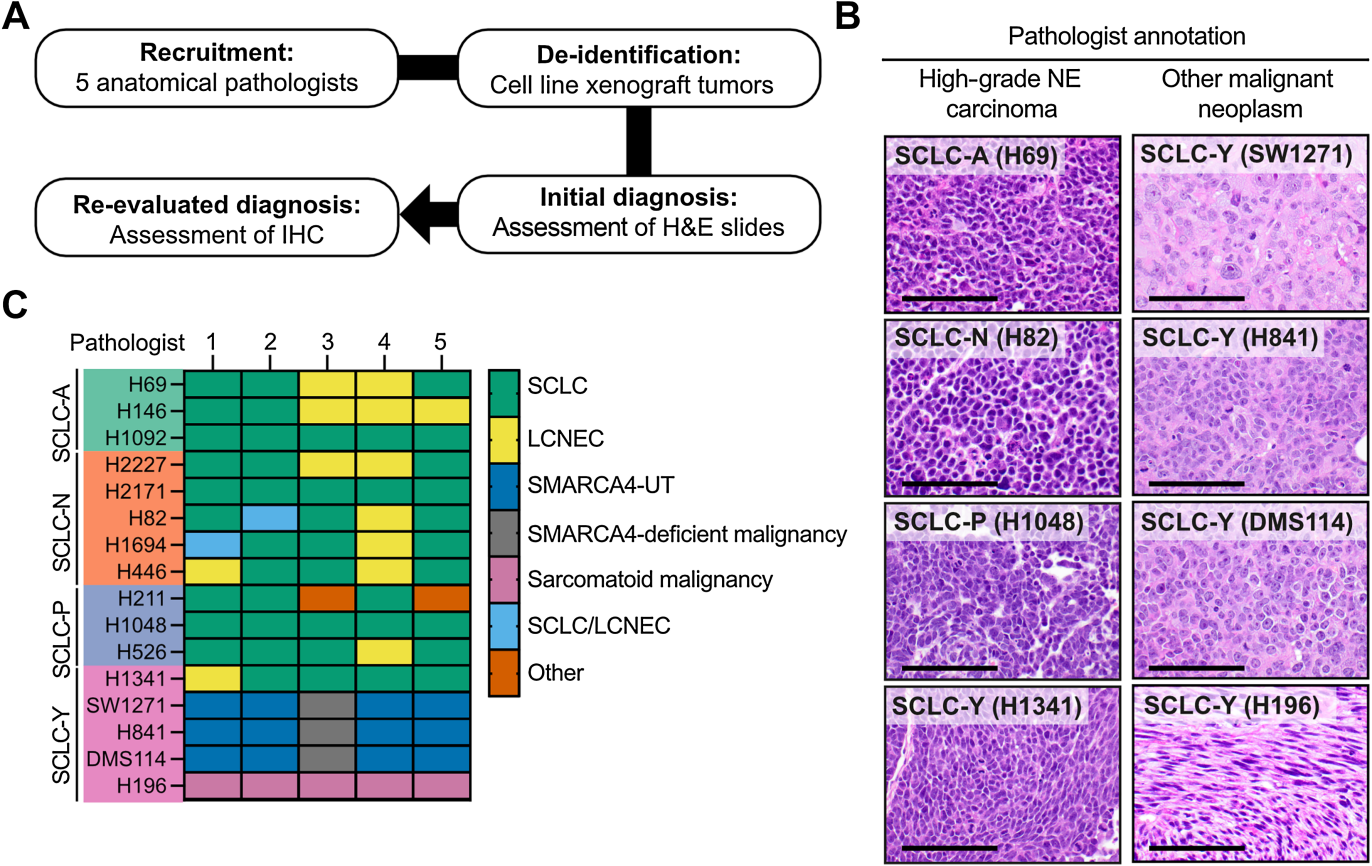
SMARCA4-deficient SCLC cell lines are morphologically and immunophenotypically similar to SMARCA4-UT. **A,** Outline of the methodology employed for assessing SMARCA4-deficient SCLC cell lines by a panel of five anatomical pathologists. **B,** Representative H&E images of cell line xenografts diagnosed as high-grade NE carcinoma (SCLC) and other malignant neoplasms (non-SCLC). Scale bar = 100 μm. **C,** Heatmap of re-evaluated diagnosis of SCLC cell line xenografts (row) and each pathologists’ classification (column). Abbreviations: LCNEC, large cell neuroendocrine cancer; SMARCA4-UT, SMARCA4-deficient undifferentiated tumor; SCLC/LCNEC, combined SCLC and LCNEC components; Other, undifferentiated tumor or small blue round cell tumor.

Inactivating mutations in *SMARCA4* are characteristic of small cell carcinoma of the ovary, hypercalcaemic type (SCCOHT) and can also occur in malignant rhabdoid tumors (24). Within the thorax, SMARCA4 loss is seen in two different settings; either in NSCLC harbouring *SMARCA4* mutations (27), or in thoracic SMARCA4-deficient undifferentiated tumors (previously named SMARCA4-deficient thoracic sarcoma; ref. 24). Although SMARCA4-deficient NSCLC (frequently adenocarcinomas) may display histologic features of de-differentiation including loss of TTF1 expression and a solid growth pattern; thoracic SMARCA4-deficient undifferentiated tumors (SMARCA4-UT) show undifferentiated round cell or rhabdoid features and reduced expression of epithelial markers such as cytokeratins and claudin-4. To better define the identity of *SMARCA4*-mutant SCLC-Y tumors, we established a panel of SCLC xenograft models derived from cell lines representative of all SCLC transcriptional subtypes, including three *SMARCA4*-mutant SCLC-Y lines. As SCLC diagnosis is primarily based on morphologic assessment of the tumor (28), five pathologists who routinely report thoracic pathology were asked to independently evaluate the de-identified H&E slides and indicate whether the tumor morphology was consistent with SCLC (Fig. 2A). Although there was some variability in individual pathologist assessments for SCLC-A, -N, and -P tumors, there was near complete consensus that the H&E appearances of four of five SCLC-Y tumors were not consistent with SCLC (Fig. 2B; Supplementary Figs. S2A and S2B).

**Figure 3.**
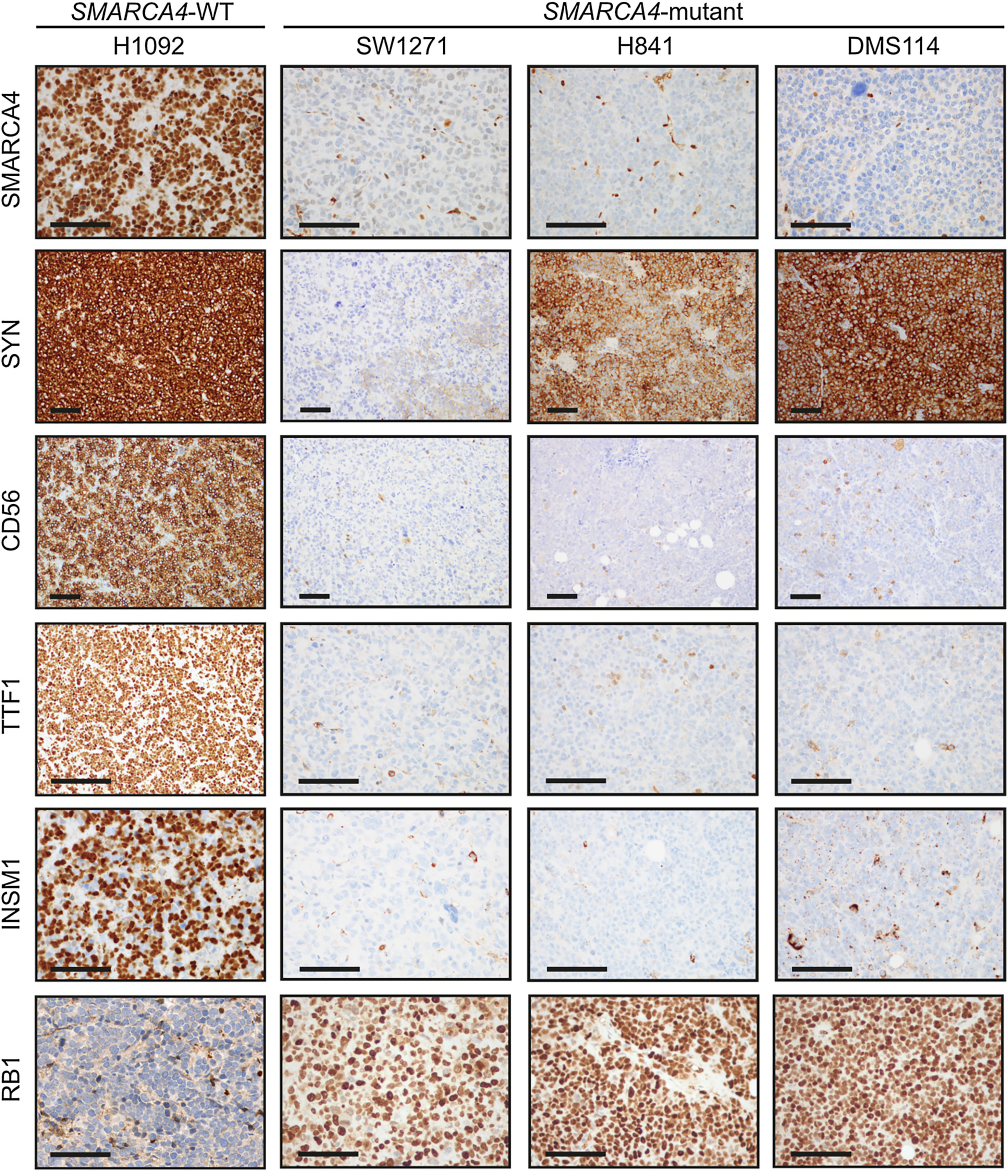
Distinct immunophenotype of SMARCA4-mutant compared with SMARCA4-WT SCLC cell lines. Representative IHC images of SMARCA4, synaptophysin (SYN), CD56, TTF1, INSM1, and RB1 in *SMARCA4*-WT, NE-high SCLC cell line (H1092) compared with *SMARCA4*-mutant SCLC cell lines. Scale bar = 100 μm.

Pathologists were then provided with an IHC panel encompassing standard diagnostic markers of thoracic malignancies (Supplementary Table S2) and asked to provide an updated diagnosis (Fig. 2C). All three of the SMARCA4-deficient SCLC-Y xenograft tumors (SW1271, H841, and DMS114) were favoured by pathologists to represent either SMARCA4-UT or SMARCA4-deficient NSCLC, rather than SCLC (Fig. 2C; ref. 15). IHC for SMARCA4 confirmed that these three SCLC-Y tumors harboring *SMARCA4* mutations were SMARCA4-deficient, whereas SMARCA4 protein expression was retained in all other tumors (Fig. 3; Supplementary Table S2). In contrast to other SCLC subtypes, these SMARCA4-deficient SCLC-Y tumors showed expression of RB1, weak cytokeratin staining and isolated expression of synaptophysin in the absence of other neuroendocrine markers INSM1 and CD56 (Fig. 3; Supplementary Fig. S3A). This IHC profile would be highly unusual for SCLC but is characteristic of thoracic SMARCA4-UT (29). We observed high concordance between neuroendocrine marker mRNA and protein expression (Supplementary Fig. S3B). Of the two SMARCA4 proficient SCLC-Y lines (H1341 and H196), only H1341 was considered morphologically and immunophenotypically consistent with SCLC (Fig. 2B and C; Supplementary Fig. S3C). Conversely, all five pathologists independently designated H196 to be a sarcomatoid (spindle cell) malignancy (Fig. 2B and C), with a differential diagnosis including sarcomatoid carcinoma, sarcoma, malignant mesothelioma, and melanoma.

To further validate the identity of the SCLC-Y lines, we reviewed the data in CCLE and compared it to independent DNA-sequencing, RNA-sequencing (RNA-seq) and signature-based histologic analysis of the same tumor lines undertaken at UT Southwestern Medical Center (UTSW; Supplementary Table S4). This comparison demonstrated high concordance between independent sequencing datasets but revealed that two of the eight previously annotated SCLC-Y lines (H1339 and H2286) were derived from tumors with a primary histologic diagnosis of NSCLC tumors rather than SCLC (30). The H157DM (*TP53*^mut^, *RB1*^wt^, *SMARCA4*^truncating^) was previously thought to be the SCLC line H1339 but was more recently identified to be the lung squamous cell carcinoma line H157, an identity that was confirmed on comparison with UTSW data (Supplementary Table S4). In addition, H2286 (*TP53*^mut^, *RB1*^wt^, *SMARCA4*^missense^) was derived from a tumor that showed mixed histology and was classified as an adenocarcinoma by UTSW according to an adenocarcinoma-squamous cell carcinoma RNA-seq signature (30). Thus, in addition to the five SCLC-Y lines directly examined in this study, these findings suggest that an additional two previously designated SCLC-Y lines (H1339/H157DM and H2286) are *SMARCA4*-mutant NSCLC rather than SCLC. On review of the origin of the final SCLC-Y line H1341, this line was derived from a biopsy of a cervical tumor in 26-year-old female. Importantly, this patient had no lung tumor on CT scan and there was no evidence of disease spread outside the cervix. This tumor was therefore diagnosed as a primary small cell carcinoma of the cervix (31). Consistent with our pathological evaluation of the H1341 xenograft (Fig. 2B; Supplementary Fig. S2A), the morphologic appearances of small cell neuroendocrine carcinomas (SCNEC) of the cervix can be indistinguishable from SCLC (32). However, cervical SCNEC tumors are typically associated with high-risk human papillomavirus (HPV) infection and have a distinct molecular profile compared with SCLC, with a lower frequency of *TP53* and *RB1* mutations (33). In keeping with a diagnosis of cervical SCNEC, H1341 lacks mutations in *TP53* and *RB1* and instead possesses mutations frequently seen in cervical SCNEC including a gain-of-function mutations in *PIK3CA* (E524K) and a *PTEN* deletion (22, 33–35). Furthermore, H1341 is positive for HPV (https://www.atcc.org/products/crl-5864, accessed October 31, 2023). Interestingly, H1341 also possesses a *SMARCB1* deletion, which has previously been reported in cervical SCNEC (34). Taken together, our findings demonstrate that all cancer cell lines originally used to classify the SCLC-Y subtype show clinical, molecular, morphologic, and/or transcriptomic features that are inconsistent with a diagnosis of SCLC (Table 1).

**Table 1.** Revised tumor classification for previously designated SCLC-Y lines.

Cell line	DepMap ID	Revised tumor classification	Age/sex	Smoking status	NE-score (CCLE RNA-seq)	*TP53*	*RB1*	*SMARCA4*
H1341	ACH-000129	Small cell neuroendocrine carcinoma of the cervix	26/F	Smoker	0.2294	WT	WT	WT
DMS114	ACH-000530	SMARCA4-UT	68/M	Unknown	0.1365	**T**	WT	**T**
SBC5	ACH-000670	SMARCA4-UT	65/M	Unknown	−0.2384	**M**	WT	**T**
H841	ACH-000292	SMARCA4-UT	51/M	Smoker	−0.3397	**M**	WT	**T**
H2286	ACH-000912	Adenocarcinoma	57/F	Smoker	−0.6746	**T**	WT	**M**
H157DM^a^	ACH-000921	Squamous cell carcinoma	59/M	Unknown	−0.7356	**T**	WT	**T**
H196	ACH-000752	Sarcomatoid malignancy	68/M	Nonsmoker	−0.7438	**M**	**T**	WT
SW1271	ACH-000890	SMARCA4-deficient malignancy	69/M	Unknown	−0.8004	**M**	WT	**M**

Abbreviations: F, female; M, male; WT, wild type; T, truncating mutation; M, missense mutation.

^a^H157DM was previously named H1339 in CCLE.

### SMARCA4-deficient SCLC cell lines have a similar transcriptome to SMARCA4-UT

**Figure 4.**
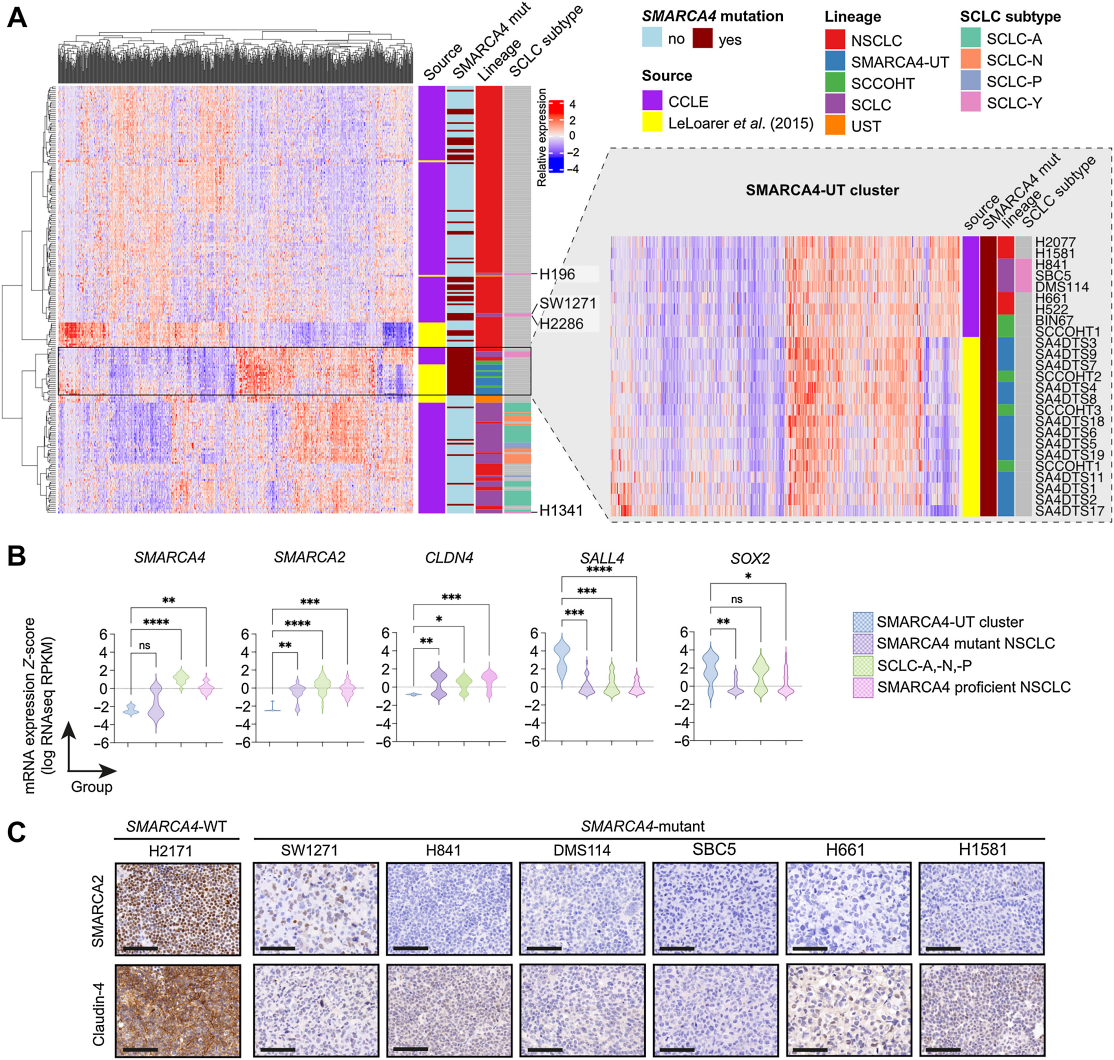
SCLC-Y cell lines with SMARCA4 loss share a similar transcriptome to SMARCA4-UT. **A,** Unsupervised hierarchical clustering, based on differentially expressed genes in primary SMARCA4-UT compared with primary *SMARCA4*-mutant NSCLC identified by Le Loarer and colleagues (24; *n* = 758 genes), was performed on SCCOHT and lung carcinoma samples [cell lines from the CCLE and bulk-RNA sequenced patient samples from Le Loarer and colleagues (24)]. Inset: SMARCA4-UT cluster from **A** with cell line and sample annotation. **B,** mRNA expression of *SMARCA4*, *SMARCA2*, and *CLDN4* (claudin-4) in cell lines of the SMARCA4-UT cluster, SMARCA4 mutant NSCLC, SMARCA4 proficient NSCLC, and SCLC-A, -N, -P (Supplementary Table S5). Kruskal–Wallis test with a Dunn multiple comparison test; *, *P* < 0.05; **, *P* < 0.01; ***, *P* < 0.001; ****, *P* < 0.0001; ns, *P* ≥ 0.05. **C,** IHC of SMARCA2 and claudin-4 in *SMARCA4*-WT and *SMARCA4*-mutant cell line xenografts. Scale bar = 100 μm.

*SMARCA4* and *SMARCA2* encode mutually exclusive core ATPase subunits of SWI/SNF chromatin remodeling complexes (26, 27). In contrast to SMARCA4-deficient NSCLC in which SMARCA2 is essential for survival (36), SMARCA4-UT and SCCOHT typically exhibit concurrent silencing of *SMARCA2* expression (29, 37). Disruption of SWI/SNF function in these tumors leads to broad transcriptional dysregulation and remarkably this epigenetic reprogramming appears to be a more dominant driver of phenotype than the tissue of origin. Thus, the transcriptome of thoracic SMARCA4-UT more closely resembles SCCOHT than SMARCA4-deficient NSCLC (24). To investigate whether the transcriptional profile of *SMARCA4*-mutant SCLC-Y lines are closely related to SMARCA4-UT or to other SCLC subtypes, we integrated RNA sequencing data from all SCLC, NSCLC, and SCCOHT cell lines in CCLE with RNA-seq data from primary tumors, including primary thoracic SMARCA4-UT, SCCOHT, and unclassified thoracic sarcomas (24). Unsupervised hierarchical clustering using a previously defined set of differentially expressed genes in primary SMARCA4-UT compared with SMARCA4-deficient NSCLC (24) revealed clustering of three SMARCA4-deficient SCLC-Y lines (H841, DMS114, SBC5) with primary thoracic SMARCA4-UT and SCCOHT (Fig. 4A). SBC5 had a similar immunophenotype (Supplementary Fig. S4) to *SMARCA4*-mutant SCLC-Y cell lines H841, DMS114, and SW1271 (Fig. 3). Conversely, the other SMARCA4-deficient SCLC-Y lines (SW1271 and H2286) clustered with *SMARCA4*-mutant NSCLC rather than SCLC (Fig. 4A). This observation was replicated through principal component (PC) analysis (Supplementary Fig. S5A), with the PC2 dimension found to be influenced by the data source (i.e., CCLE or ref. 24; Supplementary Fig. S5B). Using the top 50 genes from PC1, PC3, and PC4 for unsupervised hierarchical clustering using the top 50 genes from PC1, PC3, and PC4 confirmed the clustering of *SMARCA4*-mutant SCLC-Y cell lines with SMARCA4-UT and SCCOHT samples (Supplementary Fig. S5C).

In addition to the SCLC-Y lines, four *SMARCA4*-mutant NSCLC lines (H522, H2077, H1581, H661) localized to the SMARCA4-UT/SCCOHT cluster (Fig. 4A). The primary tumors from which the H1581 and H661 cell lines were derived, lacked morphologic features of differentiated adenocarcinoma or squamous cell carcinoma (Supplementary Fig. S4). Furthermore, all the SMARCA4-deficient lung cancer cell lines localising to this SMARCA4-UT cluster showed a gene expression profile characteristic of SMARCA4-UT, which was distinct from that of SCLC and *SMARCA4*-mutant NSCLC (29). This included SMARCA4/SMARCA2 co-deficiency, low Claudin-4, variable expression of stem cells markers SALL4, SOX2, and CD34, synaptophysin expression and absent INSM1 and NCAM1 expression (Fig. 4B and C; Supplementary Fig. S6A). Taken together, our findings demonstrate that six lines all coming from thoracic malignancies and previously designated as SCLC, harbor *SMARCA4* mutations and display histologic, and a lack of neuroendocrine features, consistent with either SMARCA4-deficient NSCLC or thoracic SMARCA4-UT rather than SCLC.

### SMARCA4-UT express YAP1

**Figure 5.**
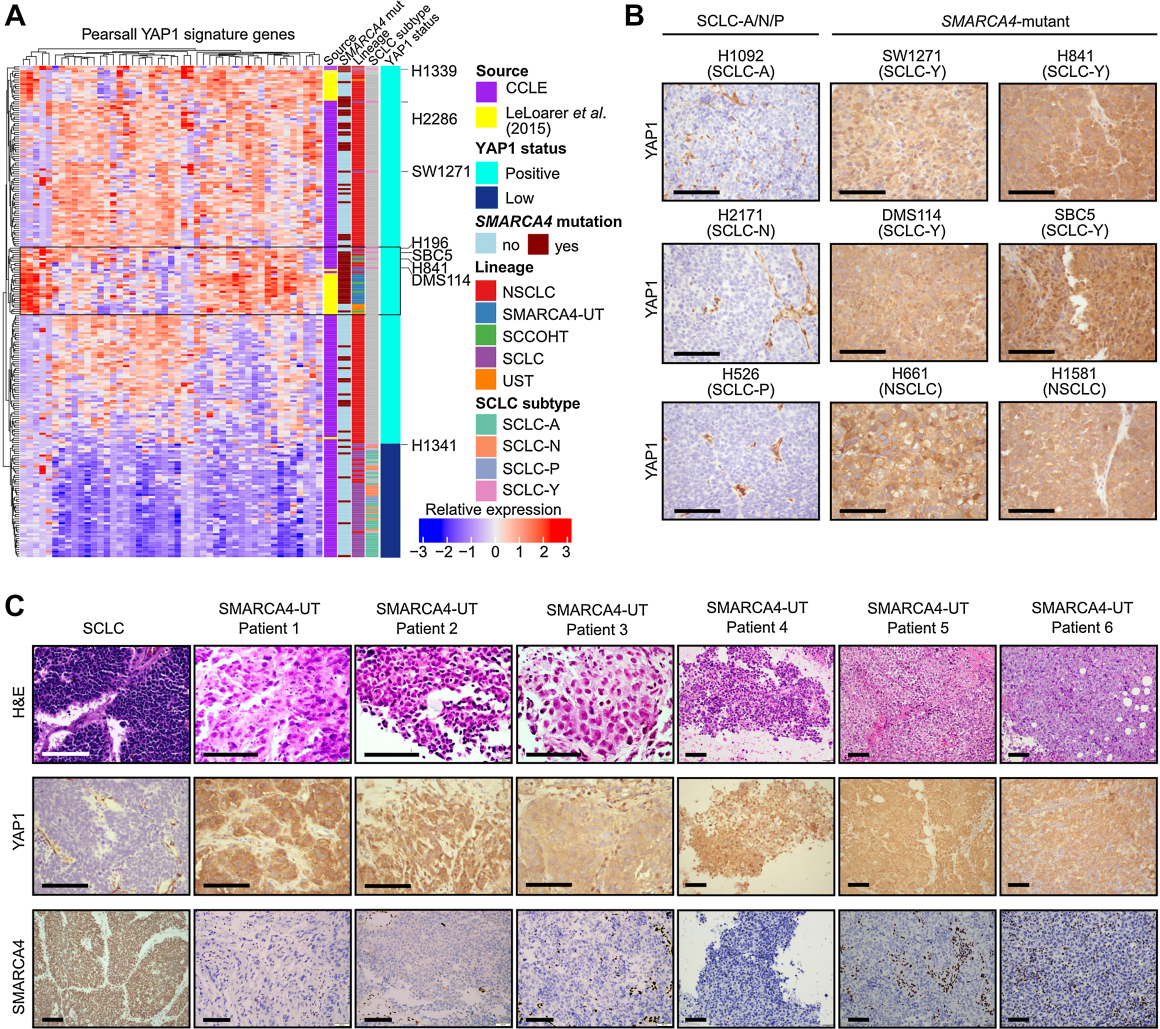
YAP1 is expressed in SMARCA4-UT primary samples. **A,** Unsupervised hierarchical clustering, based on a SCLC-specific YAP1 signature (*n* = 49 genes) identified by Pearsall and colleagues (10), was performed on SCCOHT and lung carcinoma samples [cell lines from the CCLE and bulk-RNA sequenced patient samples from Le Loarer and colleagues (24)]. Cell lines that cluster with the SMARCA4-UT samples are highlighted in the gray box. **B,** YAP1 IHC on SCLC-A/N/P and *SMARCA4*-mutant cell line xenografts. Scale bar = 100 μm. **C,** H&E, SMARCA4, and YAP1 IHC in primary SMARCA4-UT samples (*n* = 6) together with an SCLC patient sample. Scale bar = 100 μm.

Although transcriptional expression of YAP1 was initially used to stratify the SCLC-Y subtype (2), IHC staining of SCLC patient samples has largely failed to support YAP1 as a defining molecular marker in SCLC (8). To first correlate YAP1 mRNA and protein expression, we performed YAP1 IHC on *SMARCA4*-mutant (SW1271, H841, DMS114, H661, and H1581) and *SMARCA4*-wildtype (H2171, H1092, and H526) cell line xenografts. Consistent with the high YAP1 mRNA expression seen in SCLC-Y cell lines (2), strong YAP1 protein expression was observed in all tumor cell lines within the SMARCA4-UT cluster and NSCLC cluster (Fig. 5A). Interestingly, the cervical SCNEC line H1341 showed patchy expression of YAP1 and had the strongest neuroendocrine marker expression of the previously designated SCLC-Y lines (Supplementary Fig. S6B; Table 1).

To validate whether our findings in cell line xenografts are representative of primary SMARCA4-UT tumors in patients, we initially made use of a YAP1 signature, previously used to interrogated YAP1 expression in SCLC Circulating Tumor Xenograft (CTX) models (10). As expected, SCLC cell lines representing the -A, -N, and -P subtypes exhibited low YAP1 signature expression and displayed a distinct clustering pattern (Fig. 5B). Conversely, *SMARCA4*-mutant SCLC-Y cell lines clustered with primary thoracic SMARCA4-UT and SCCOHT patient samples (Fig. 5B; Supplementary Table S9; Fig. 5B). Moreover, *YAP1* mRNA expression was comparable between SMARCA4-UT and *SMARCA4*-mutant NSCLC (Supplementary Fig. S6A), consistent with the proposed oncogenic role of YAP1 in NSCLC (38). To validate these findings at the protein level, we performed IHC staining for YAP1 on primary SMARCA4-UT patient samples (*n* = 6; Fig. 5C). Indeed, in addition to displaying a characteristic undifferentiated morphology with loss of SMARCA4 expression, SMARCA4-UT patient material showed strong expression of YAP1 (Fig. 5C). Critically, this was in stark contrast to the lack of YAP1 expression seen in a primary SCLC tumor, where high YAP1 expression is detected specifically in endothelial and stromal cells (Fig. 5C; ref. 8). Our findings demonstrate that, in contrast to SCLC (8), both xenografts of SMARCA4-mutant cell lines previously classified as SCLC-Y and patient-derived primary SMARCA4-UT tumor samples show diffuse, uniformly strong YAP1 protein staining. Thus, altogether our findings indicate that diffuse YAP1 expression is a consistent feature of SMARCA4-UT, explaining how previous erroneous annotation of both SMARCA4-UT cell lines and YAP1-positive NSCLC lines as SCLC, led to the grouping of these lines within a distinct YAP1-positive SCLC subgroup.

## Discussion

Understanding SCLC heterogeneity and the distinct biology of the proposed SCLC subtypes is critical for the development of targeted therapies for this devastating disease. Here, we shed light on the origins of the proposed controversial “SCLC-Y” subtype, making the surprising observation that the majority of SCLC-Y tumor cell lines, on which the classification of SCLC-Y category was largely based, harbour *SMARCA4* mutations. Detailed molecular and histopathological characterisation of these tumors revealed that these tumors show features in keeping with SMARCA4-deficient malignancies rather than SCLC.

Several of the SCLC-Y tumor lines showed close transcriptional similarities with primary thoracic SMARCA4-UT. These tumors showed pathologic features consistent with SMARCA4-UT and gene expression profiles consistent with this diagnosis, including loss of both SMARCA4 and SMARCA2 expression and low claudin-4. In contrast to SCLC-A, -N, and -P subtypes, these SMARCA4-deficient SCLC-Y tumors retained RB1 expression and expressed synaptophysin, but lacked both CD56 and INSM1, a profile that is characteristic of SMARCA4-UT. Interestingly, *SMARCA4* inactivating mutations were recently detected in 1.5% of SCLC patient samples (39). Although these tumors also harbored *TP53* and *RB1* genetic alternations, importantly accompanying histopathology was not available, thus the inclusion of SCLC mimics, such as SMARCA4-UT could not be excluded.

The classification of SCLC subtypes was based both on primary SCLC samples as well as SCLC cell lines, however, cell lines were particularly enriched within the SCLC-Y category (2). All of these lines were developed between 1975 and 1991 from patients clinically diagnosed as having SCLC, in nearly every case by pathologists recognized as world experts in SCLC diagnosis working as part of a group with extensive experience in SCLC clinical trials (NCI-Navy Medical Oncology Branch; ref. 40). Nevertheless, these diagnoses were made well before our current knowledge of the key genomic features and IHC markers characteristic of SCLC and other lung tumors, such as SMARCA4 deficient malignancies. Thoracic SMARCA4-UT is a recognized mimic of SCLC, and 23% of SMARCA4-UT in a recent series were initially diagnosed as small cell or large cell neuroendocrine carcinoma (29). Like SCLC, SMARCA4-UT typically occurs in middle-aged smokers and can mimic several histological features of SCLC including small cell morphology, high proliferation index, crush artefact, and synaptophysin expression (29). These features may account for the original SCLC diagnosis, however the morphology and IHC profile of the SMARCA4-deficient SCLC-Y xenografts were unanimously considered by a panel of pathologists to be consistent with thoracic SMARCA4-UT or SMARCA4-deficient carcinoma as opposed to SCLC. Although we did not identify *SMARCA4* mutations in two of the eight SCLC-Y lines, one of these lines was pathologically not consistent with SCLC and the other was derived from a 26-year-old patient diagnosed with a primary small cell carcinoma of the cervix rather than SCLC. Thus, altogether we found little evidence to support a diagnosis of SCLC for any of the SCLC-Y lines tested (Table 1).

The expression of YAP1 is inversely correlated with the expression of neuroendocrine markers in SCLC, and thus “classical” neuroendocrine high SCLC lacks expression of YAP1. In contrast, YAP1 is expressed in NSCLC, including adenocarcinoma, squamous cell carcinoma, and a proportion of large cell neuroendocrine carcinomas (8, 41). Importantly, here we also demonstrate that primary SMARCA4-UT universally exhibit diffuse and strong expression of YAP1 protein. The expression of YAP1 in multiple lung malignancies therefore complicates the use of YAP1 expression to define a specific subtype of SCLC, particularly given the occurrence of combined tumors in which YAP1 expressing NSCLC may be admixed with SCLC, and the existence of YAP1-positive tumors that can mimic SCLC histologically, such as basaloid squamous cell carcinoma, poorly differentiated adenocarcinoma, high-grade adenoid cystic carcinoma, and SMARCA4-UT.

Altogether, our findings suggest that, unlike ASCL1, NEUROD1, and POU2F3, YAP1 is not a subtype defining transcription factor in SCLC. This is consistent with a recent study in a patient cohort, which failed to identify a distinct YAP1 expressing SCLC subtype (8). Although focal YAP1 expression has been detected in some primary SCLC samples (11), our findings together with recent studies in SCLC xenograft and GEM models suggests that this is due to intratumoral heterogeneity (9, 10, 13, 42). In this context, classical ASCL1 driven SCLC can transition to a “neuroendocrine low” phenotype, which is associated with expression of YAP1. This phenotypic plasticity is a feature of *RB1* null SCLC, and the emergence of neuroendocrine low, YAP1 expressing cells has been associated with chemoresistance, activation of Notch signaling, and expression of mesenchymal and inflammation-associated genes (10, 13, 42).

Importantly, our findings demonstrate that the patient-derived cancer cell lines initially used to define the SCLC-Y subtype actually represent SMARCA4-UT, NSCLC, or other SCLC mimics, and therefore are not representative models of “triple negative” or “inflamed” SCLC. We also stress that a subset of human SCLC tumors exhibit a triple A/N/P negative phenotype, low neuroendocrine marker expression and a more “inflamed” gene signature. However, there is little evidence to date supporting the use of YAP1 as a reliable marker for these tumors. Although the majority of CCLE cell lines defined as SCLC-Y appear to be SMARCA4-deficient malignancies, this is likely because these tumors were diagnosed historically, without access to immunohistochemical and molecular testing used today and before SMARCA4-deficient lung malignancies were a recognized disease entity. We therefore do not anticipate that “triple negative” or “SCLC-I” tumors identified in recent, thoroughly histopathologically characterized SCLC cohorts are likely to represent SMARCA4-UT. However, our work highlights the importance of comprehensive histopathologic and molecular characterization of SCLC tumors subtyped in clinical cohorts, with particular focus on excluding potential SCLC mimics for tumors that lack neuroendocrine marker expression or have an unusual molecular background, for example retained expression of wild-type RB.

SMARCA4-UT is a highly aggressive lung malignancy for which effective treatments are needed. Furthermore, these tumors exhibit shared transcriptomic and phenotypic features with aggressive SMARCA4-deficient malignancies occurring at other sites, including SSCOHT and malignant rhabdoid tumor. SMARCA4-UT typically present as a large central thoracic tumor involving the pulmonary hilum and/or mediastinum in young to middle-aged smokers. Histologically the tumor consists of sheets of variably discohesive epithelioid cells, which typically have prominent nucleoli and frequently show focal rhabdoid morphology. SMARCA4-UT lack clear evidence of epithelial differentiation and the characteristic diagnostic IHC profile is weak or absent expression of epithelial markers such as Cytokeratins and Claudin-4, negative or focal TTF-1 or p40 staining, and loss of SMARCA4 expression. In addition, some cases show expression of stem cell markers such as CD34, SOX2, or SALL4 (15). Approximately 70% of SMARCA4-UT show IHC staining for synaptophysin but other neuroendocrine markers such as CD56 and INSM1 are typically negative (29).

Given the broad use of the SCLC-Y cell lines we now identify to be SMARCA4-UT in therapeutic studies, our findings provide new insight into potential therapeutic vulnerabilities in SMARCA4-deficient malignancies. For example, the IL-15 super-agonist, N-808 (43), arginine deprivation (44), and inhibition of Aurora kinase B (45), checkpoint kinase 1 (CHK1; ref. 46) have all demonstrated anticancer activity in SMARCA4-deficient SCLC-Y cell lines. Furthermore, findings from preclinical studies employing these SMARCA4-deficient SCLC-Y cell lines have been used as a basis to initiate clinical trials in SCLC, highlighting the clinical importance of resolving the identity of these tumors (47, 48). In contrast to SCLC, emerging evidence suggests that SMARCA4-UT may respond poorly to chemotherapy (49, 50). Conversely, several of the SCLC-Y lines that we now identify to be SMARCA4-UT have previously been shown to be sensitive to CDK4/6 inhibitors (25), and consistent with this SMARCA4-deficiency, has been shown to be a strong predictor of sensitivity to CDK4/6 inhibitors in NSCLC and SCCOHT tumor models (51, 52). The cell lines we have identified as SMARCA4-UT have higher expression of immune and MHC antigen presentation genes than classical neuroendocrine SCLC-A and SCLC-N, raising the possibility that these tumors may respond to immunotherapy. Interestingly, there are several reports of SMARCA4-UT exhibiting responses to immune checkpoint blockade with anti-PD-1 or anti-PD-L1 therapy (53, 54).

Taken together, these SMARCA4-deficient cell lines previously characterized as SCLC-Y may serve as patient derived preclinical models of SMARCA4-UT to accelerate the discovery of new therapeutics for this aggressive malignancy.

## Supplementary Material

Supplementary Figure S1 Characterisation of SMARCA4 mutations in lung cancer cell lines from the CCLE.

Supplementary Figure S2 Initial histopathological evaluation of SCLC xenografts.

Supplementary Figure S3 SCLC-specific markers are weak/lost in SMARCA4-deficient SCLC-Y cell lines.

Supplementary Figure S4 H&E and immunohistochemistry of cell line xenografts H661, H1581 and SBC5.

Supplementary Figure S5 SMARCA4-UT cluster validation.

Supplementary Figure S6 mRNA expression of SMARCA4-UT associated genes and YAP1 IHC.

Supplementary Table S1 Immunohistochemistry antibodies, protocols and evaluation criteria.

Supplementary Table S2 Immunohistochemistry scoring table.

Supplementary Table S3 Clinical profiles and immunohistochemistry of SMARCA4-UT patients.

Supplementary Table S4 Comparison of sequencing data between CCLE and UTSW.

Supplementary Table S5 CCLE cell lines used in Figure 4 and Supplementary Figure S6A.

Supplementary Table S6 List of cell lines from Figure 1D and their NE-score.

Supplementary Table S7 26 mutated genes unique to SCLC-Y cell lines.

Supplementary Table S8 Other mutations and copy number alterations associated with SCLC-Y.

Supplementary Table S9 SMARCA4-UT cell lines and patient samples based on unsupervised hierarchical clustering using YAP1 signature genes.
