# Supplementary material for: Molecular and Pathologic Characterization of YAP1-Expressing Small Cell Lung Cancer Cell Lines Leads to Reclassification as SMARCA4-Deficient Malignancies
Source: Clin Cancer Res. 2023 Dec 7;30(9):1846–58. doi: 10.1158/1078-0432.CCR-23-2360 (PMC11061608; doi:10.1158/1078-0432.CCR-23-2360)
Supplement: Supplementary Figure S1 — Characterisation of SMARCA4 mutations in lung cancer cell lines from the CCLE. [file ccr-23-2360_supplementary_figure_s1_suppsf1.pdf]

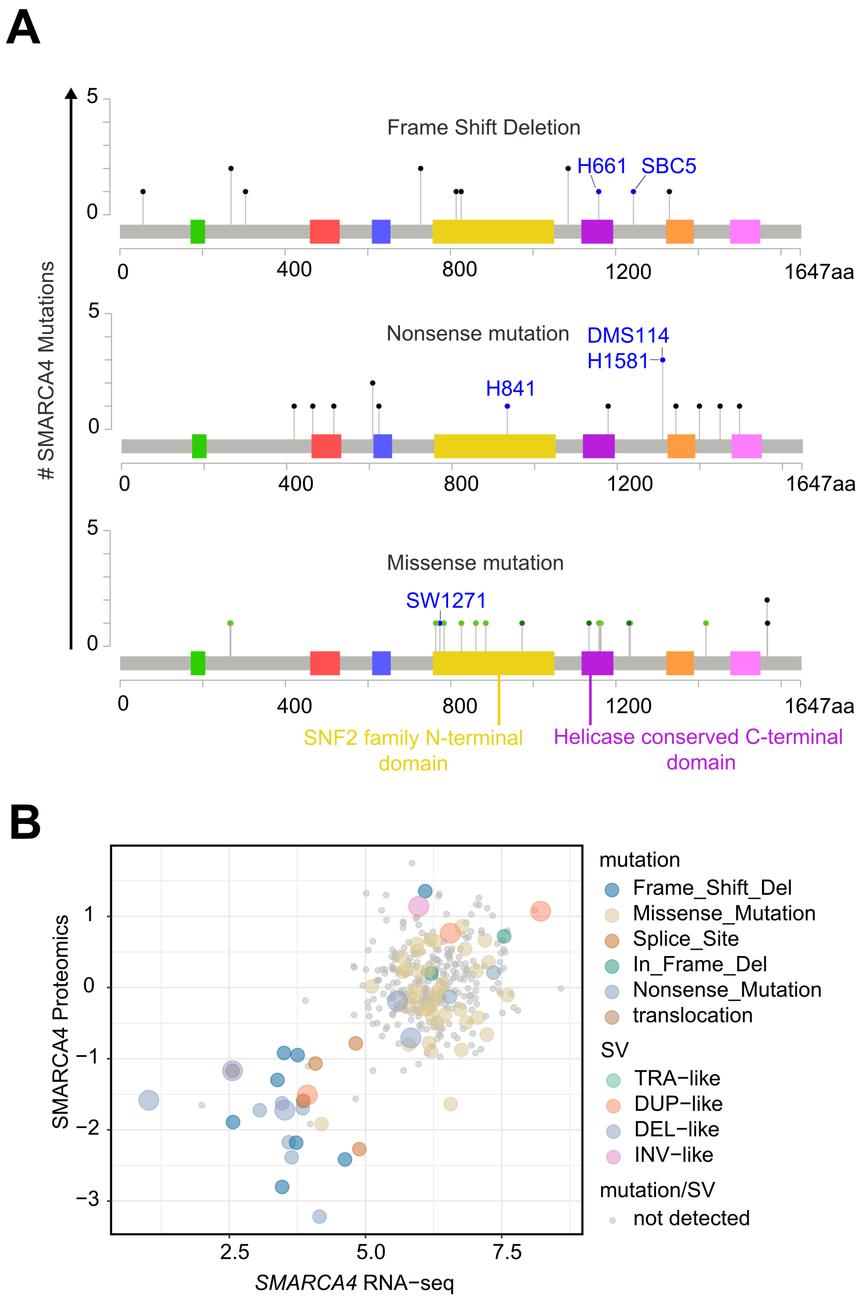

Supplementary Figure S1.

Characterisation of SMARCA4 mutations in lung cancer cell lines from the CCLE. **A**, Lollipop diagram showing the position of frame shift deletions, nonsense and missense mutations in *SMARCA4* across lung cancer cell lines. *SMARCA4*-mutant SCLC-Y cell lines are annotated in blue. **B**, Key mutations associated with a decrease in SMARCA4 protein and mRNA include frame shift deletions, nonsense mutations, deletion-like structural variations and splice-site mutations. Most missense mutations do not result in a decrease in SMARCA4 protein nor mRNA.
