# Supplementary material for: Molecular and Pathologic Characterization of YAP1-Expressing Small Cell Lung Cancer Cell Lines Leads to Reclassification as SMARCA4-Deficient Malignancies
Source: Clin Cancer Res. 2023 Dec 7;30(9):1846–58. doi: 10.1158/1078-0432.CCR-23-2360 (PMC11061608; doi:10.1158/1078-0432.CCR-23-2360)
Supplement: Supplementary Figure S2 — Initial histopathological evaluation of SCLC xenografts. [file ccr-23-2360_supplementary_figure_s2_suppsf2.pdf]

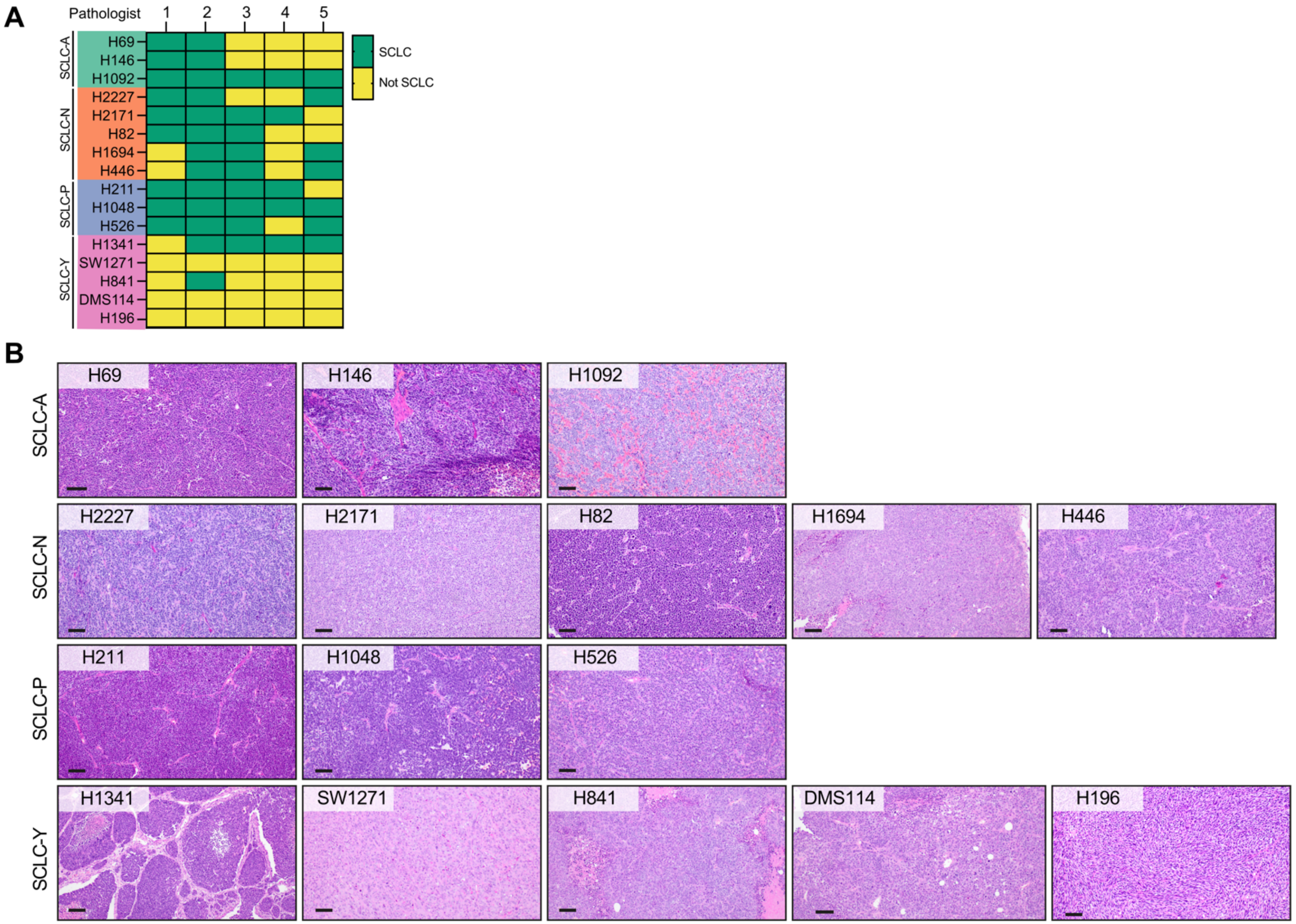

Supplementary Figure S2.

Initial histopathological evaluation of SCLC xenografts. **A**, Heatmap of initial diagnosis (based on H&E) of SCLC cell line xenografts (row) and each pathologists' classification (column). SCLC favoured diagnosis (green) and other non-SCLC malignancies (yellow) are highlighted. **B**, Representative H&E images of SCLC cell line xenografts used in this study. Scale bar=100 µm.
