# Supplementary material for: Molecular and Pathologic Characterization of YAP1-Expressing Small Cell Lung Cancer Cell Lines Leads to Reclassification as SMARCA4-Deficient Malignancies
Source: Clin Cancer Res. 2023 Dec 7;30(9):1846–58. doi: 10.1158/1078-0432.CCR-23-2360 (PMC11061608; doi:10.1158/1078-0432.CCR-23-2360)
Supplement: Supplementary Figure S3 — SCLC-specific markers are weak/lost in SMARCA4-deficient SCLC-Y cell lines. [file ccr-23-2360_supplementary_figure_s3_suppsf3.pdf]

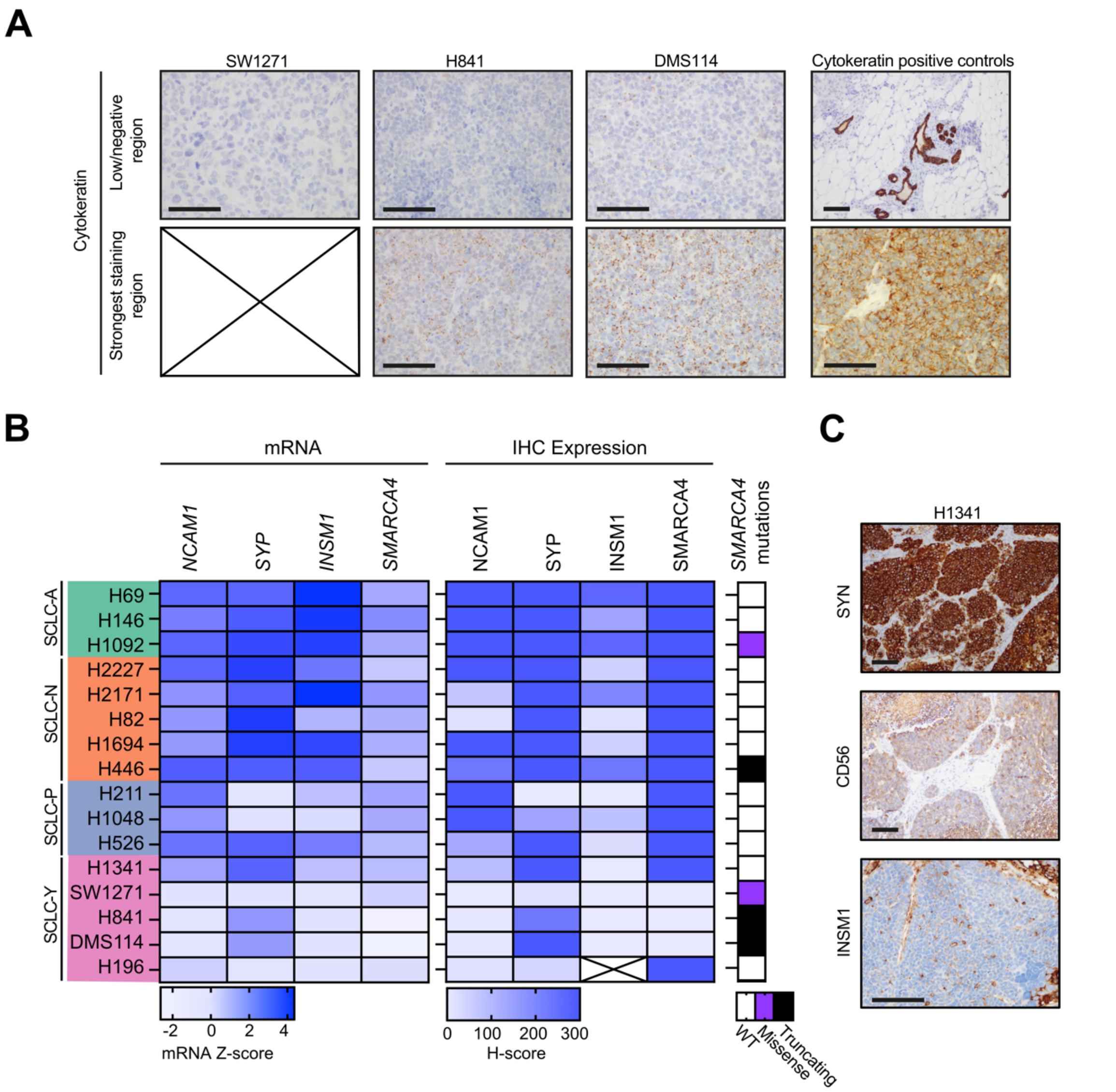

Supplementary Figure S3.

SCLC-specific markers are weak/lost in SMARCA4-deficient SCLC-Y cell lines. **A**, Cytokeratin staining in SMARCA4-deficient SCLC-Y cell lines DMS114, SW1271 and H841 showing weak cytokeratin staining relative to the cytokeratin positive controls (right panel). Cytokeratin staining for SW1271 was consistently low/negative. Scale bar=100 µm. **B**, mRNA expression of neuroendocrine markers (*NCAM1, SYP, INSM1*) and *SMARCA4* compared protein levels evaluated by immunohistochemistry. The INSM1 immunophenotyping in H196 was not interpretable due to a high level of non-specific background staining. **C,** Immunohistochemistry staining of SCLC-Y cell line xenograft H1341 with NE markers (SYN, CD56 and INSM1). Scale bar=100 µm.
