# Supplementary material for: Molecular and Pathologic Characterization of YAP1-Expressing Small Cell Lung Cancer Cell Lines Leads to Reclassification as SMARCA4-Deficient Malignancies
Source: Clin Cancer Res. 2023 Dec 7;30(9):1846–58. doi: 10.1158/1078-0432.CCR-23-2360 (PMC11061608; doi:10.1158/1078-0432.CCR-23-2360)
Supplement: Supplementary Figure S4 — H&E and immunohistochemistry of cell line xenografts H661, H1581 and SBC5. [file ccr-23-2360_supplementary_figure_s4_suppsf4.pdf]

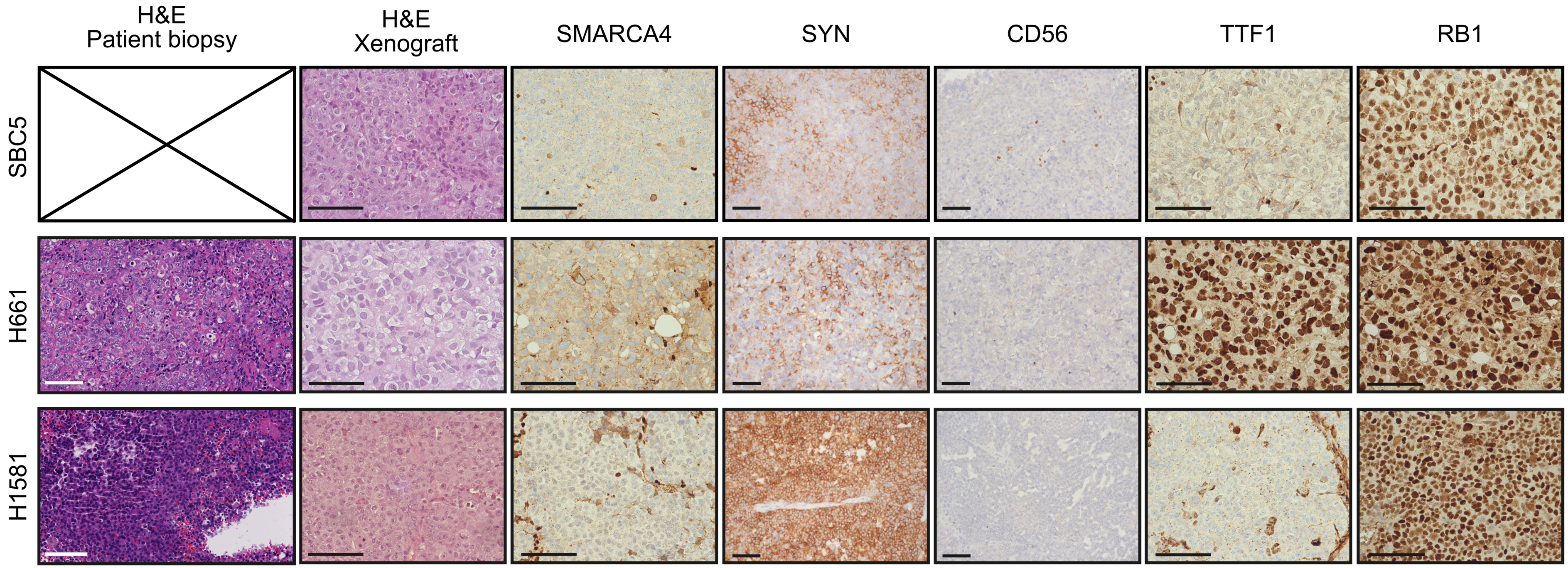

Supplementary Figure S4.

H&E and immunohistochemistry of cell line xenografts H661, H1581 and SBC5. The H&E from the original patient biopsy H661 and H1581 are derived from is also displayed. The original patient biopsy for SBC5 was not available. Scale bar=100 µm.
