# Supplementary material for: Molecular and Pathologic Characterization of YAP1-Expressing Small Cell Lung Cancer Cell Lines Leads to Reclassification as SMARCA4-Deficient Malignancies
Source: Clin Cancer Res. 2023 Dec 7;30(9):1846–58. doi: 10.1158/1078-0432.CCR-23-2360 (PMC11061608; doi:10.1158/1078-0432.CCR-23-2360)
Supplement: Supplementary Figure S5 — SMARCA4-UT cluster validation. [file ccr-23-2360_supplementary_figure_s5_suppsf5.pdf]

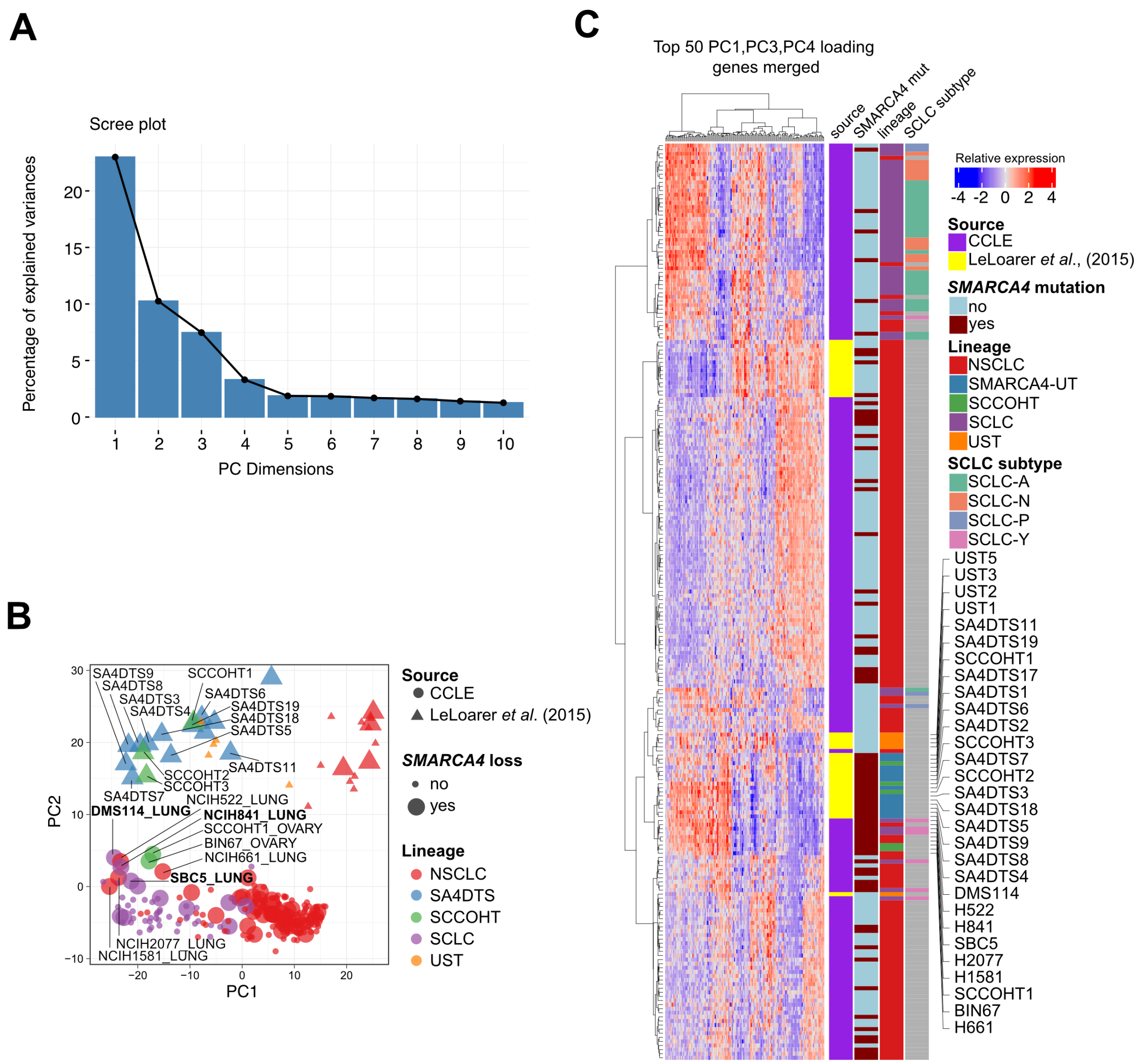

Supplementary Figure S5.

SMARCA4-UT cluster validation. **A,** The Scree plot displays the top four PC dimensions, which accounts for 44% of the total variance. **B**, Samples from CCLE and Le Loarer *et al.* separate on PC2, suggesting genes correlated with PC2 is associated with batch effects between cell line and bulk-sequenced tumour samples. SMARCA4 loss was determined by integrating genetic aberration and RNA/protein expression data. For the expression data, we used model-based clustering implemented by R package *mclust* (1) to determine SMARCA4-high and SMARCA4-low cell lines. For proteomics data we used the feature for "sp|P51532|SMCA4_HUMAN". We then merged the two sets of SMARCA4 loss status data. A loss status of "yes" is assigned to a cell line with any mutation or low expression found in any of the datasets, a loss status of "no" is assigned to a cell line with no mutation found and not a SMARCA4-low line based on any of the expression data. Abbreviations: NSCLC=Non-

small cell lung cancer; SADTS=SMARCA4-deficient thoracic sarcoma (SMARCA4-UT); SCCOHT=Small cell carcinoma of the ovary hypercalcaemic type; UST=Unclassified thoracic sarcoma. **C**, Principal component (PC) analysis of CCLE and tumour samples was performed using the top 50 genes with high loadings from PC1, PC3, and PC4, we created a smaller gene set (*n*=150) to cluster samples from CCLE and primary tumours from Le Loarer *et al*.(25). Unsupervised hierarchal clustering showed a similar clustering pattern to the heatmap in Fig. 3A, confirming a closer relationship of SCLC-Y lines (DMS114, H841 and SBC5) with SMARCA4-UT than SCLC.

(1) Scrucca L, Fop M, Murphy TB, Raftery AE. mclust 5: Clustering, Classification and Density Estimation Using Gaussian Finite Mixture Models. R J. 2016;8:289–317.
