# Supplementary material for: Molecular and Pathologic Characterization of YAP1-Expressing Small Cell Lung Cancer Cell Lines Leads to Reclassification as SMARCA4-Deficient Malignancies
Source: Clin Cancer Res. 2023 Dec 7;30(9):1846–58. doi: 10.1158/1078-0432.CCR-23-2360 (PMC11061608; doi:10.1158/1078-0432.CCR-23-2360)
Supplement: Supplementary Figure S6 — mRNA expression of SMARCA4-UT associated genes and YAP1 IHC. [file ccr-23-2360_supplementary_figure_s6_suppsf6.pdf]

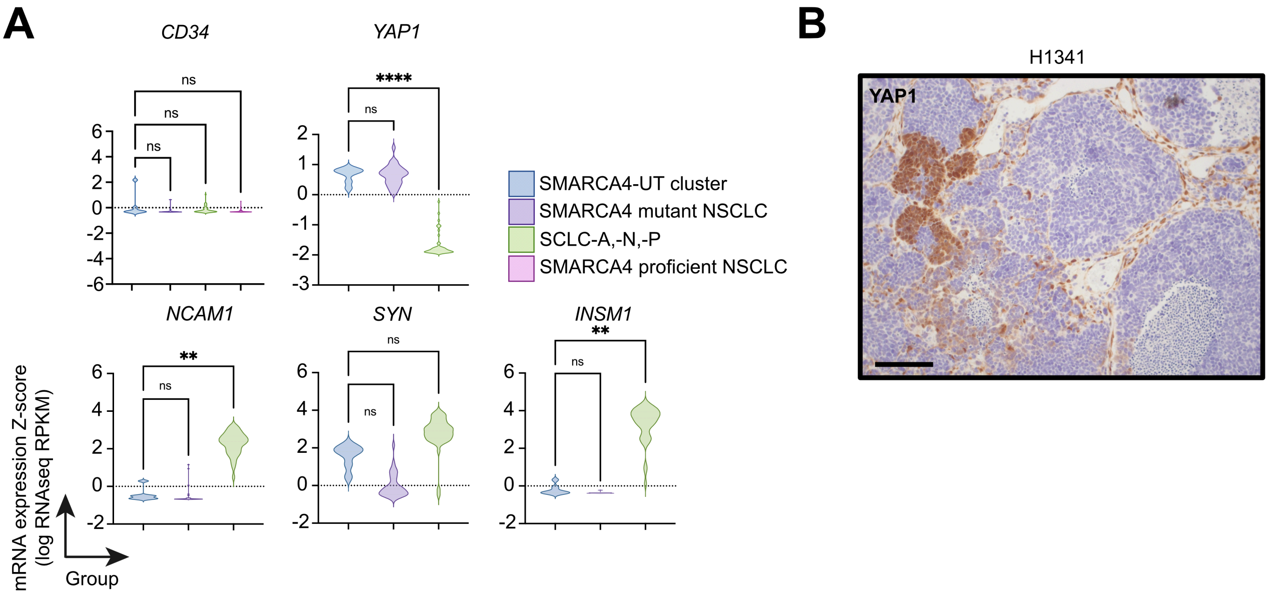

Supplementary Figure S6.

mRNA expression of SMARCA4-UT associated genes and YAP1 IHC. **A**, mRNA expression of clinical markers of SMARCA4-UT (*CD34*), *YAP1* and neuroendocrine markers (*NCAM1*, *SYN* and *INSM1*) in cell lines of the SMARCA4-UT cluster, SMARCA4 mutant NSCLC, SMARCA4 proficient NSCLC and SCLC-A, -N, -P. Kruskal-Wallis test with a Dunn's multiple comparison test * $p < 0.05$, ** $p < 0.01$, *** $p < 0.001$, **** $p < 0.0001$, ns $p \geq 0.05$. **B**, Immunohistochemistry of YAP1 on H1341. Scale bar=100 µm.
